# Mitochondria: Insights into Crucial Features to Overcome Cancer Chemoresistance

**DOI:** 10.3390/ijms22094770

**Published:** 2021-04-30

**Authors:** Ilaria Genovese, Marianna Carinci, Lorenzo Modesti, Gianluca Aguiari, Paolo Pinton, Carlotta Giorgi

**Affiliations:** 1 Department of Medical Sciences, Section of Experimental Medicine, University of Ferrara, 44121 Ferrara, Italy; ilaria.genovese@unife.it (I.G.); marianna.carinci@unife.it (M.C.); lorenzo.modesti@unife.it (L.M.); paolo.pinton@unife.it (P.P.); 2 Department of Neuroscience and Rehabilitation, Section of Biochemistry, Molecular Biology and Genetics, University of Ferrara, 44121 Ferrara, Italy; gianluca.aguiari@unife.it

**Keywords:** mitochondrial dynamics, mitochondrial Ca^2+^ homeostasis, metabolic plasticity, drug resistance, cancer

## Abstract

Mitochondria are key regulators of cell survival and are involved in a plethora of mechanisms, such as metabolism, Ca^2+^ signaling, reactive oxygen species (ROS) production, mitophagy and mitochondrial transfer, fusion, and fission (known as mitochondrial dynamics). The tuning of these processes in pathophysiological conditions is fundamental to the balance between cell death and survival. Indeed, ROS overproduction and mitochondrial Ca^2+^ overload are linked to the induction of apoptosis, while the impairment of mitochondrial dynamics and metabolism can have a double-faceted role in the decision between cell survival and death. Tumorigenesis involves an intricate series of cellular impairments not yet completely clarified, and a further level of complexity is added by the onset of apoptosis resistance mechanisms in cancer cells. In the majority of cases, cancer relapse or lack of responsiveness is related to the emergence of chemoresistance, which may be due to the cooperation of several cellular protection mechanisms, often mitochondria-related. With this review, we aim to critically report the current evidence on the relationship between mitochondria and cancer chemoresistance with a particular focus on the involvement of mitochondrial dynamics, mitochondrial Ca^2+^ signaling, oxidative stress, and metabolism to possibly identify new approaches or targets for overcoming cancer resistance.

## 1. Introduction

Cancer remains one of the greatest public health issues worldwide. According to the National Cancer Institute (NIH), the incidence between 2013 and 2017 was 442.4 per 100,000 men and women per year, with a death rate of 158.3 per 100,000 men and women per year (https://www.cancer.gov/about-cancer/understanding/statistics, accessed on 3 March 2021). The interest in developing novel and successful therapies is ardent and continuous; nevertheless, this pursuit still suffers some failures, such as the rise in chemoresistance and multidrug resistance (MDR).

MDR represents the major cause of treatment failure and cancer relapse after surgical removal. To date, two types of chemoresistance development mechanisms have been reported: intrinsic and acquired [1,2]. Intrinsic chemoresistance, as the name suggests, is an innate characteristic of cancer cells due to preexisting resistance-mediating factors in the tumor mass that impede the effectiveness of the treatment even before it is administered. On the other hand, acquired resistance, which develops after treatment administration, is generally caused by mutations or alterations in the adaptive response machinery that span from the increased expression of therapeutic targets, inactivation of drugs, overexpression of transporters for drug extrusion (mdr pumps), promotion of DNA damage repair, and metabolic changes to the activation of alternative survival pathways [3].

An additional feature of this quite intricate scenario is cell heterogeneity in the bulk cancer; indeed, this permits the coexistence of diverse cell populations. Recent findings have outlined that cancer stem cells (CSCs) may be the pioneers of innate/acquired [4,5,6] conferring and spreading a resistance phenotype in the whole cancer pool and to other organs [3,4,7,8].

Even if CSCs are a small percentage (less than 1–2%) [9,10] of the cancer population, they have been found in leukemias (such as Acute Myeloid Leukemia [AML]) as in solid tumors (such as breast, lung, brain, prostate, colon, liver and head and neck cancers) [11,12], and generally, their relative abundance is associated with the clinical outcome [13]. As CSCs have stemness features, they are more prone to last in a quiescent state than canonical cancer cells; this is why most of the current treatments that target dividing cells have very little effect on CSCs [14].

As mentioned above, cancer cells with acquired drug resistance may exhibit metabolic changes, whereas CSCs are characterized by great metabolic plasticity that gives them the ability to survive in adverse conditions such as hypoxia [15,16,17]. Mitochondria play a central role in the bridge between metabolism and tumor progression, as they can decide cell fate [18]. Interestingly, it has been observed that CSCs increase the expression of genes encoding mitochondrial proteins [19,20], highlighting the crucial importance of mitochondria for cancer cell survival, resistance, and spreading.

In recent years, there has been growing interest regarding mitochondria as potential crucial targets for cancer therapy [21], mainly because mitochondria can rapidly adapt to stressful conditions to permit cell survival. In fact, mitochondria are involved in metabolic changes and survival pathways regulating the oxidative stress response, mitochondrial plasticity, known as mitochondrial dynamics (mitochondrial fusion, mitochondrial fission, mitochondrial transfer, mitophagy) and Ca^2+^ homeostasis [22,23,24,25,26,27], as they are the site where all these processes are often strictly intertwined.

Therefore, it seems that mitochondrial plasticity is a key feature in many steps of tumorigenesis, especially chemoresistance. Indeed, recent research suggests that in chemoresistant ovarian cancer cells, the mitochondrial bioenergetic switch to oxidative metabolism represents an advantage with respect to their non-resistant counterparts, and this shift occurs along with relevant mitochondrial remodeling [28]. Another recent study showed that the transfer of healthy mitochondria from fibroblasts to HeLa cells or SAS r^0^ cells (depleted of mitochondrial DNA [mtDNA]) restored their proliferative capacity and sensitivity to cisplatin treatment, suggesting that mitochondrial transfer can be considered a potential therapeutic strategy [29].

With this review, we aimed to gather all the recent findings regarding mitochondrial dynamics and homeostasis to outline their roles in cancer chemoresistance. A deeper understanding of these features could be crucial to overcoming the limitations that chemotherapeutic treatments have in many cancer types to pave the way towards more successful therapies.

## 2. Role of Mitochondrial Remodeling (Fusion, Fission, Mitophagy, and Transfer) in Cancer Chemoresistance

**Figure 1:**
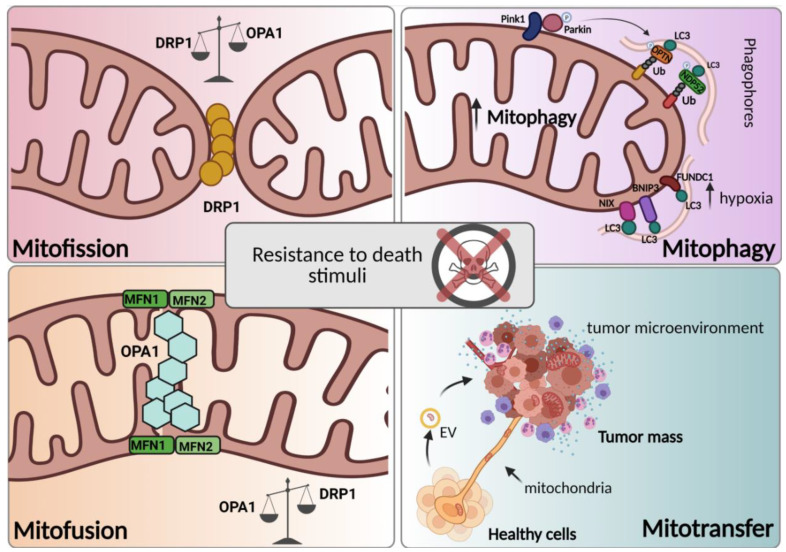
Mitochondrial dynamics in chemoresistance. Mitotransfer, mitophagy, and mitochondrial fusion and fission (respectively, mitofusion and mitofission) are processes related to mitochondrial dynamics. These processes, depending on their activation timing, tumor subtype, and microenvironment, can foster chemoresistance. The details of the processes are reviewed in the paragraph. (Created with Biorender.com, accessed on 18 March 2021).

Mitochondrial activities such as fusion, fission, mitophagy, and mitochondrial transfer make mitochondria highly dynamic organelles [30]. The fine-tuned regulation of mitochondrial dynamics allows the maintenance of functional mitochondria through the control of their shape, number, quality, and distribution in cells. In addition, to ensure correct mitochondrial functions, these coordinated dynamic transitions are essential for responding to cellular requirements via rapid morphological adaptation to the metabolic state of the cell [31]. Since many functions of mitochondria involved in cell homeostasis are closely linked to their morphology [32], it is not surprising that dysregulation of mitochondrial dynamics is related to several pathologies ranging from neurodegenerative diseases [33] to ischemic stroke [34] and cancers [35,36]. In particular, impaired mitochondrial dynamics have been associated with the initiation and progression of several cancer types and cancer metastasis, CSC survival, and drug resistance [37,38] (Figure 1), thus suggesting that targeting mitochondrial dynamics is a potential therapeutic strategy for fighting cancer. In this section, we critically reviewed the involvement of mitochondrial dynamics in cancer chemoresistance.

### 2.1. Mitochondrial Fusion and Mitochondrial Fission in Cancer Chemoresistance

Two opposing processes, mitochondrial fusion and fission, according to the cellular metabolic requirements, allow mitochondria to constantly divide or connect to each other to form networks or fragments, respectively [31,39]. Mitochondrial fusion and fission are important in a variety of cell functions, including the cell cycle [40], developmental processes [41], and apoptosis [42]. Moreover, different functions of mitochondria are reflected in their structure. While fusion is commonly associated with high energy demand, resulting in a hyperfused mitochondrial network with increased ATP production and protection against autophagy [43,44,45,46,47]; fission is mainly linked to apoptosis, facilitating the segregation of mtDNA upon mitosis and eliminating defective mitochondria through selective isolation of parts of the organelle from the network [48,49,50]. In other words, pro-survival signals are correlated with elongated mitochondria, while cell death is connected to fragmented mitochondria. 

However, depending on the cell’s state, fusion and fission can be proapoptotic or antiapoptotic, complicating their role in cancer [51,52].

Mitochondrial fusion results from the union of two distinct mitochondria into one through a process that requires the coordination of three dynamin-related GTPases, optic atrophy 1 (OPA1) and mitofusin (MFN) 1 and 2 [53,54]. Fusion of the outer mitochondrial membrane (OMM) is mainly carried out by the formation of homotypic and heterotypic oligomers of MFN1 and MFN2, which leads to membrane clustering in a GTP-dependent manner [55,56], whereas OPA1, at IMM, requires MFN1 to mediate mitochondrial fusion [54,57]. In contrast to MFN1 and MFN2, OPA1 requires proteolytic cleavages for activation. The OPA1 precursor is imported into the mitochondrion, where it undergoes removal of the N-terminal mitochondrial targeting sequence by peptidases, thus producing the long isoform of OPA1 (l-OPA1) that is embedded in the IMM. Further processing of l-OPA1 in the matrix is mediated by different proteases to form the OPA1 short isoform (s-OPA1). OMA1 is required for OPA1 cleavage, which occurs at basal levels but is strongly induced by mitochondrial depolarization [58,59,60], while the intermembrane (i-AAA) protease YME1L is involved in cleavage at the S2 site of OPA1 [61]. The absence of OMA1 in cells results in the inhibition of stress-induced OPA1 processing, affecting both mitochondrial fusion and fission. In this case, l-OPA1 is stabilized, and the formation of s-OPA1 isoforms is prevented, resulting in the maintenance of a tubular mitochondrial network and protection against apoptosis [62]. The combination of long and short Opa1 isoforms is mandatory for fusion, although only the long isoform has been demonstrated to be fusion competent [60,63]. Additionally, accelerated OPA1 proteolysis has been reported to trigger mitochondrial fragmentation [64]. A relevant role of mitochondrial fusion in chemoresistance is now emerging; indeed, it has been reported that chemoresistant ovarian cancer cells have more interconnected mitochondrial networks than their chemosensitive counterparts [65,66]. Consistently, in the absence of OMA1 or p53, two proteins involved in OPA1 processing, chemoresistant gynecologic cells, show fused mitochondria even in response to cisplatin, reflecting their inability to undergo fragmentation when OPA1 processing is altered [67]. Interestingly, prolonged treatment with venetoclax in AML cells results in drug resistance. In particular, venetoclax-resistant AML cells show upregulation of OPA1, which likely establishes resistance to cytochrome c release upon stimulation [68]. In accordance with the role of mitochondrial fusion in chemoresistance, MFN2 and oxidative phosphorylation (OXPHOS) have been found to be significantly upregulated in surviving leukemia cells since the knockout of MFN2 substantially increases Jurkat sensitivity to doxorubicin [69]. In addition, it has been found that the inhibition of mitochondrial fusion by silencing MFN1 increases cisplatin sensitivity in human neuroblastoma cells [70]. All these findings indicate that mitochondrial fusion strictly relies on the adaptation of metabolic changes by promoting cell survival, increasing ATP production, and decreasing apoptosis and mitochondrial fragmentation, which may provide a potential target for increasing chemotherapy efficacy. However, further investigation unraveling the links between mitochondrial fusion and cell sensitivity to chemotherapy is necessary to develop therapies offering improvement in clinical outcomes.

As stated before, mitochondrial fusion is balanced by opposing fission events. Mitochondrial fission is characterized by the division of one mitochondrion into two mitochondria and primarily requires the action of the master regulator of mitochondrial fission, dynamin-related protein 1 (DRP1), which is a cytosolic guanosine triphosphatase (GTPase) that is recruited to the OMM of mitochondria by outer membrane receptors, including fission protein homolog 1 (FIS1), mitochondrial fission factor (MFF), and mitochondrial dynamics proteins (MIDs) 49/51, [71]. When DRP1 reaches the mitochondrial membrane, it oligomerizes and wraps around the constriction points of dividing mitochondria, promoting mitochondrial fission [72]. Of note, several post-translational modifications regulate the role of DRP1 in mitochondrial fragmentation in response to specific fission stimuli, especially phosphorylation. DRP1 phosphorylation is regulated by a variety of kinases, including PKCδ [73], Cdk1/cyclin B [49], ERK1/2 [74], Ca^2+^/calmodulin-dependent protein kinase I alpha (CaMKIalpha) [75], adenosine monophosphate (AMP)–activated protein kinase (AMPK) [76], and cyclic AMP-dependent protein kinase (PKA) [77]. Although mitochondrial fission represents the opposite process of mitochondrial fusion, increasing evidence shows an important role of the first process in chemoresistance as well [78]. Interestingly, latent membrane protein 1 (LMP1), a major Epstein–Barr virus (EBV)-encoded oncoprotein, has been found to regulate DRP1 through two oncogenic signaling axes: AMPK and cyclin B1/Cdk1. Thus, in EBV-LMP1-positive nasopharyngeal carcinoma (NPC), mitochondrial fission mediated by DRP1 phosphorylation promotes cell survival and cisplatin resistance. This finding strongly suggests that targeting DRP1 could be a promising therapeutic strategy to overcome resistance in this kind of cancer. Indeed, metformin or cucurbitacin E, which interferes with the DRP1 upstream kinase AMPK or cyclin B1/Cdk1, increased the chemosensitivity of NPC cells to cisplatin [79]. Although several works reported the implication of different fission-independent signaling pathways in chemoprotection following the interactions between leukemia cells and stromal cells [80,81,82], ERK/DRP1-dependent mitochondrial fission has also been shown to be involved in bone marrow-derived mesenchymal stem cell (MSC)-induced drug resistance in T-cell acute lymphoblastic leukemia cells [83,84].

Hypoxia promotes mitochondrial fission and cisplatin resistance in ovarian cancer cells via ROS; consistently, suppression of mitochondrial fission by Mdivi-1, a putative DRP1 inhibitor, or DRP1 silencing enhanced the cisplatin sensitivity of hypoxic ovarian cancer cells. Therefore, the mitochondrial fission of cancer cells adapting to the hypoxic tumor microenvironment could be a potential target for chemoresistant cells [66,85]. Moreover, although Mdivi-1 is widely reported to inhibit Drp1-dependent fission, recent studies highlighted that the compound acts by reversibly inhibiting complex I as well, thus modifying mitochondrial ROS production, possibly contributing to the observed effects [86]. In addition, it has been reported that Mdivi-1 impaired cell proliferation, also acting on oxidative metabolism [87], highlighting that DRP1-inhibition remains an ongoing challenge. Conversely, it has been reported that increased levels of DRP1 induced by ABT737, an inhibitor of antiapoptotic BCL-2/BCL-XL, promote mitochondrial fission, leading to apoptosis and mitophagy in ovarian cancer cells resistant to cisplatin. Accordingly, Mdivi-1 weakened ABT737-induced processes. Thus, targeting antiapoptotic BCL-2 family proteins may be an emerging therapeutic strategy for patients with cisplatin-resistant ovarian cancer since it allows the induction of DRP1-dependent apoptotic mitochondrial fission [66,88].

Chemoresistance is common in most patients with colorectal cancer. Notably, chemotherapeutic drugs that promote the release of high-mobility group box 1 protein (HMGB1) from dying cells elicit ERK1/2-mediated DRP1 phosphorylation via its receptor for the advanced glycation end product (RAGE). All of these events trigger the autophagy process, inducing chemoresistance and regrowth of the surviving cancer cells after treatment. Administration of an HMGB1 inhibitor or a RAGE blocker abolished DRP1 phosphorylation, enhancing sensitivity to chemotherapeutic treatment by inhibiting autophagy [89]. Another study also elucidated the role of ERK-induced DRP1 phosphorylation in the chemoresistance of bladder cancer cells. The study reported that LASS2 inhibits bladder cancer invasion and chemoresistance through the regulation of ERK-DRP1-induced mitochondrial fission [90].

Since mitochondrial fusion and fission represent adaptive cellular systems to cellular metabolic requirements, multiple mechanisms of the mitochondrial dynamics regulation could represent cancer strategies for developing chemoresistance in a context-dependent manner.

Accordingly, anticancer drugs promoting the release of HMGB1, in colorectal cancers, or increased ROS production and hypoxic conditions in ovarian cancers, are commonly associated with fission-driven chemoresistance. On the contrary, time-dependent exposure to drug in AML, loss of function of p53 in ovarian cancers, increased levels of MFN2 and OXPHOS in response to doxorubicin in Jurkat leukemia cells are usually associated with fusion-driven chemoresistance. Thus, mitochondrial fusion or fission, although opposite processes, depending on the cancer type and on the hypoxic or metabolic state, can be both promoters of cancer chemoresistance. Depending on the conditions, the induction or inhibition of mitochondrial fusion or fission could help in fighting cancer chemotherapy resistance. However, more insights into cellular fusion and fission strategies on the basis of chemoresistance have to be further investigated.

### 2.2. Mitophagy in Cancer Chemoresistance

In addition to mitochondrial fusion and fission, proper mitochondrial function is also ensured by an important degradative process involved in mitochondrial quality control and mitophagy. Several studies have shown that mitochondrial fission coordinates with mitophagy, representing a prerequisite to the mitochondrial degradation pathway in many mammalian cell types [91,92]. The term mitophagy outlines the selective degradation of damaged mitochondria in a process involving the formation of a double-membrane structure called an autophagosome, which sequesters the organelle, allowing its autophagosomal degradation following fusion with a lysosome [93]. Essentially, the mitophagy pathway can be divided into two types: ubiquitin-mediated mitophagy and receptor-mediated mitophagy. The most well-studied ubiquitin-mediated pathway involved in cancer chemoresistance is the PINK1-Parkin pathway. Mitochondrial impairment or depolarization leads to the accumulation of phosphatase and tensin homolog (PTEN)-induced kinase 1 (PINK1) at the OMM. Here, the kinase recruits and phosphorylates the E3 ligase Parkin, promoting its E3 ligase activity [94,95,96]. Once activated, Parkin can ubiquitylate several OMM proteins that are then recognized by ubiquitin-dependent receptors, also known as autophagy receptors, such as p62/SQSTM1 (p62), optineurin (OPTN), NBR1 (neighbor of BRCA1), and NDP52 (nuclear dot protein 52 kDa). These receptors, which harbor both ubiquitin-binding domains and LC3-interacting regions (LIRs), bind ubiquitinated proteins and promote their degradation by mitophagy through binding to LC3 on the autophagosome membrane [97].

Several mitophagy receptors are involved in PINK-Parkin-independent mitophagy, including Bcl-2/adenovirus E1B 19 kDa interacting protein 3 (BNIP3), BNIP3-like (BNIP3L)/NIX, and FUN14 domain-containing protein 1 (FUNDC1), have been found to be associated with chemoresistance. All these receptors are OMM proteins associated with hypoxia-mediated mitophagy that bind LC3 through their own LIR [98,99,100,101].

Thus, mitophagy plays a dual role in cancer therapy; on the one hand, mitophagy can induce cancer cell death; on the other hand, it is able to promote cancer cell survival [102], indicating the complexity of mitophagy regulation in cancer. The role of autophagy/mitophagy differs in different stages of tumor development [103]. Although the cancer type and tumor microenvironment complicate the role of this degradative pathway in tumorigenesis, it is well accepted that in the early stage of cancer progression, mitophagy inhibits tumor progression, while in the latest stages of tumor development, the occurrence of mitophagy promotes cancer cell survival [102]. Importantly, the occurrence of drug resistance to common chemotherapeutic drugs, including cisplatin, doxorubicin (Dox), 5-fluorouracil (5-FU), and paclitaxel, due to autophagy or mitophagy often leads to treatment failure [104]. Therefore, an increasing number of studies highlight the involvement of the mitophagy process in chemoresistance.

High levels of PINK1 expression have been associated with a poorer prognosis in several tumor types. In non-small-cell lung cancer (NSCLC), PINK1 downregulation potentiates cisplatin-induced NSCLC cell apoptosis [105,106], and similar results have been obtained in esophageal squamous cell carcinoma (ESCC) patients undergoing neoadjuvant chemotherapy [107]. In support of the role of mitophagy in cancer chemoresistance, it has been reported that the inhibition of PINK1/Parkin-dependent mitophagy through PINK1 siRNA, mdivi-1, or bafilomycin A1 (Baf A1) promotes B5G1-induced cell death, sensitizing multidrug-resistant cancer cells to a new betulinic acid analog, B5G1 [108]. Notably, depletion of FUNDC1 in patients with cervical cancer, where high levels of FUNDC1 are associated with a poor prognostic outcome, significantly enhances cell sensitivity to cisplatin and ionizing radiation [109]. Further, in hepatocellular carcinoma (HCC), it has been reported that treatment with palliative transarterial embolization/transarterial chemoembolization (TAE/TACE) promotes survival and the acquisition of a more invasive phenotype in HCC cells [110]. The blockage of DRP1-mediated mitochondrial fission, and thus mitophagy, increases the incidence of mitochondrial apoptosis in HCC cells, suggesting a new possible approach of targeting mitophagy to enhance apoptosis in TAE/TACE-mediated ischemic hypoxic conditions [111].

In addition to the specific activation of mitophagy, cancer cell heterogeneity adds a further layer of complexity to this process with respect to cancer chemoresistance. Indeed, CSCs are known to be drug-resistant. Doxorubicin-induced mitophagy contributes to drug resistance, and BNIP3L silencing enhances the sensitivity to doxorubicin in human colorectal CSCs isolated from HCT8 cells, suggesting that mitophagy contributes to drug resistance [112]. All these findings indicate a crucial role of mitophagy in cancer chemoresistance. Thus, combining drugs targeting mitophagy with chemotherapy drugs represents an encouraging way to overcome chemoresistance and improve therapeutic outcomes in several cancer types.

### 2.3. Mitochondrial Transfer in Cancer Chemoresistance

Mitochondrial dynamics are accompanied by intercellular dynamics in a mechanism called mitochondrial transfer. In both physiological and pathological conditions, two cells respond to external signals, including chemokines and cytokines, as well as therapeutic drugs, directly transmitting biological information via horizontal transfer [113,114,115]. The physical processes of this cellular communication mechanism include the establishment of gap junctions, tunneling nanotubes (TNTs), and extracellular vesicle (EV) transport [116,117,118,119]. Among these connection mechanisms, a functional role for TNTs and EVs in several cancer types is emerging. TNTs are transient cytoplasmic extensions characterized by long actin-based fibers, with lengths of several hundred micrometers and diameters ranging from 50 to 1500 nm, that connect nonadjacent cells [116,120]. TNTs allow the trafficking of relatively large cargos, including organelles such as mitochondria, through motor-adaptor protein complexes related to the mitochondrial Rho GTPase Miro1, from a donor cell to a recipient cell [121]. EV communication is characterized by the exchange of signals, including soluble and insoluble factors, as well as structural proteins, nucleic acids, and lipids, through the release of membrane-enclosed particles termed EVs [122,123,124]. EVs include several kinds of vesicles, including exosomes (30–100 nm in diameter), microvesicles (MVs) (100–1000 nm in diameter), and a very recently identified cancer-derived EV population called oncosomes (1–10 μm in diameter) [125]. In recent decades, increasing attention has been given to intercellular communication, identifying this mechanism as a crucial factor inducing heterogeneity in the tumor microenvironment, highlighting its role in contributing to chemoresistance [113]. TNT-mediated mitochondrial transfer, first observed in mesothelioma [126,127], occurs both between cancer cells and between cancer and normal cells of the microenvironment of several cancer types [128,129,130]. An additional role of TNT in promoting drug resistance is through intercellular drug efflux, as demonstrated in chemotherapy-induced pancreatic cancer cells [113]. Functional benefits from the acquisition of mitochondria have been documented; the common result of mitochondrial transfer in recipient cells is the acquisition of a survival advantage from the mitochondrial uptake process. Indeed, mitochondria transferred by TNTs increase the OXPHOS output and ATP production of the target cells, affecting their metabolism, and as functional consequences, recipient cancer cells exhibit enhanced proliferative, migratory properties and resistance to stress [131,132,133]. Therefore, it is not surprising that a number of studies have reported that cancer cells take advantage of this mechanism to enhance their chemoresistance and increase their regrowth potential after treatment. Consistently, it has been reported that mitochondrial transfer from endothelial cells to MCF7 breast cancer cells promotes their resistance to doxorubicin [134]. Moreover, functional mitochondria derived from bone marrow stromal cells have been found to increase resistance to cytarabine treatment in AML [135]. Accordingly, it has been proposed that mitochondrial transfer, from primary bone marrow stromal cells (BMSC) to the primary AML blasts, via TNT likely contributes to chemoresistance in AML [136]. In a study on ovarian cancer and breast cancer cell lines, it was demonstrated that the preferential transfer through TNT of mitochondria was from endothelial to cancer cells, demonstrating that mitochondria uptake, inducing phenotypic advantage in recipient cells, resulted in the acquisition of chemoresistance [128]. TNT mitochondrial transfer has also been linked to human tumor-activated stromal cells (TASCs) and glioblastoma cells, conferring resistance to standard treatments (radiotherapy and chemotherapy) [137]. Drug resistance in T-ALL makes this leukemia one of the most aggressive hematologic malignancies. Interestingly, it has been demonstrated that upon the induction of oxidative stress by chemotherapeutic drugs, T-ALL cells were able to transfer mitochondria to MSCs; this process is mediated by TNTs and ICAM-1 and contributes to the cell adhesion-mediated drug resistance [138].

In addition, it has been demonstrated that MSCs transfer mitochondria to damaged neural stem cells (NSCs) via the formation of TNTs, allowing NSC survival after cisplatin treatment. Consistently, the inhibition of actin polymerization in MSCs blocks the transfer of mitochondria and abrogates the beneficial effect of MSCs on NSCs. Conversely, the enhancement of mitochondrial transfer by Miro1 overexpression further increases the survival of NSCs after cisplatin treatment [139]. In line with these studies, the horizontal transfer of mtDNA in circulating EVs from patients with hormonal therapy-resistant metastatic breast cancer promotes the exit of therapy-induced cancer stem-like cells from dormancy, leading to endocrine therapy resistance in OXPHOS-dependent breast cancer [140]. Since mitochondrial transfer clearly provides survival advantages following chemotherapy, this mechanism could represent a future therapeutic target for several chemoresistant cancer types.

## 3. Role of Proteins That Regulate Mitochondrial Ca^2+^ Homeostasis in Cancer ChemoResistance and Death Resistance: An Overview

Ca^2+^ signaling is essential for a plethora of cell functions; therefore, [Ca^2+^]_i_ must be kept under control to enable proper cell physiology. Specifically, extracellular [Ca^2+^] is greater than 1 mM, while cytosol has a [Ca^2+^] concentration of approximately 100 nM, and intracellular stores in the sarco/endoplasmic reticulum have a concentration >100 µM; this homeostasis is finely tuned and maintained by Ca^2+^ transport systems, ion channels, Ca^2+^ pumps, Ca^2+^ sensor proteins and other Ca^2+^-binding proteins that are located at the plasma membrane, endoplasmic reticulum (ER), mitochondria, or cytoplasm [141,142]. Mitochondria are crucial organelles involved in the regulation of cell Ca^2+^ homeostasis. Ca^2+^ regulates the mitochondrial respiration rate, which depends on ATP production; nevertheless, too much Ca^2+^ could also prompt mitochondrion-mediated apoptosis [143]. Cancer cell survival strictly depends on the combination of sustained mitochondrial bioenergetics together with evasion from death stimuli; to counterbalance metabolic demand and survival signals, cells have evolved mitochondrial Ca^2+^ influx and efflux systems, which have been found to be extremely helpful for tumorigenesis [144]. Due to its multifaceted role in regulating the fate between cell survival and death, mitochondrial Ca^2+^ signaling pathways are under growing investigation since the proteins involved in those pathways may represent alternative targets for cancer therapy.

Together with mitochondria, the ER plays a central role in the regulation of [Ca^2+^]_i_, specifically via a series of proteins and factors constituting membranous tethering systems named mitochondria-associated membranes (MAMs) [145,146,147,148,149,150,151,152,153,154].

In fact, mitochondria-ER crosstalk has a pivotal role in cell adaptation to stress stimuli. MAMs may play a role in response to chemotherapeutic treatment by regulating Ca^2+^ signaling, as the ER can regulate Ca^2+^ transfer not only to the mitochondria but also to the cytoplasm. Chemotherapeutic drugs cause a rapid increase in [Ca^2+^]_cyt_ [155,156]; moreover, these shifts in cytosolic Ca^2+^ are thought to be early markers of cytotoxicity in response to various oxidative stress stimuli [155,156].

However, mitochondrial Ca^2+^ homeostasis may also be affected by some chemotherapeutic drugs since these drugs can either have a direct effect on mitochondria or indirectly act on Ca^2+^ uptake by affecting mitochondrial membrane potential (ΔΨ) [157].

Furthermore, it has been shown that patient-derived mesothelioma cancer cells critically deregulate intracellular Ca^2+^ signaling, mainly due to alterations in mitochondrial Ca^2+^ uptake. This feature is correlated with evident resistance to cell death upon chemotherapeutic treatment; indeed, the restoration of proper Ca^2+^ homeostasis resulted in increased sensitivity to the drugs [158].

The relationship between mitochondrial Ca^2+^ uptake and cell death induction is not unambiguous, since Ca^2+^ uptake is necessary for the maintenance of ΔΨ as ATP is produced; however, a persistent Ca^2+^ signal towards mitochondria is perceived as a stress stimulus, thus translating into apoptosis activation (for a review on the mitochondrial Ca^2+^ issue, see [155]).

To date, the relevance of intracellular Ca^2+^ signaling in tumorigenesis and chemoresistance has undeniably been acknowledged; however, the majority of evidence takes into consideration proteins located either on the ER membrane (STIM, SERCA, IP3R2, and IP3R3) or plasma membrane (PMCAs, ORAI, TRPCs, TRPMs, TRPVs, and CACNAs) or soluble proteins (calpains, S100 family, and calmodulin), which have effects on Ca^2+^ homeostasis overall [159,160,161,162,163,164,165,166].

**Figure 2:**
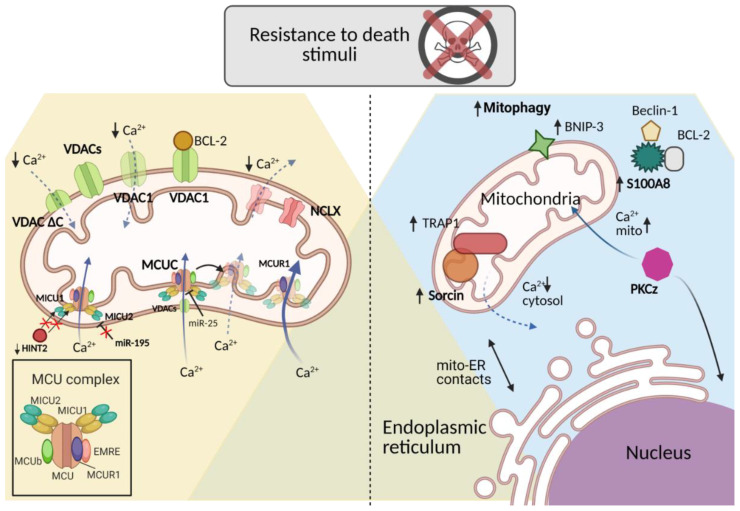
Mitochondrial Ca^2+^ related proteins in chemoresistance regulation. Mitochondrial-resident proteins and cytosolic mitochondrial-related proteins are able to regulate mitochondrial Ca^2+^ homeostasis affecting the sensitivity towards chemotherapeutic treatments. The mechanisms of regulation of mitochondrial Ca^2+^ homeostasis in relation to different cancer types and treatments are detailed in the paragraph. (Created with Biorender.com, accessed on 18 March 2021).

The purpose of this chapter is to provide new insights into the role of some of the most critical mitochondrion-resident or -associated proteins involved in the regulation of tumor drug resistance through Ca^2+^ signaling, pointing towards potential novel investigation targets (Figure 2).

### 3.1. Mitochondrial Membrane Proteins

The contribution of mitochondria to the chemoresistant phenotype may be summarized in two ways: (i) mitochondrial ATP production is necessary for the activity of mdr pumps (mdr1 and mdr4), also known as P-glycoproteins (P-gp), that are ATP-binding cassette family members involved in the active extrusion of xenobiotics outside the cells (see introduction); (ii) defective mitochondrial outer membrane permeabilization (MOMP) or impaired activation/opening of the mitochondrial permeability transition pore (mPTP), that is involved in the release of proapoptotic factors, such as cytochrome c [167].

For these reasons, the ion channels residing both in the OMM and IMM may impact resistance to death stimuli, since OMM channels participate in the permeabilization process, while the IMM channels regulate the maintenance and adaptation of ΔΨ, thus influencing the efficiency of mitochondrial respiration and ROS production [168,169].

Next, we critically review the most important proteins in both the OMM and IMM, outlining their implications in resistance to death stimuli in cancer cells.

#### 3.1.1. The Mitochondrial Ca^2+^ Uniporter (MCU) Complex

The MCU complex (MCUC) is a macromolecular complex consisting of regulatory and pore-forming subunits [170]; the pore consists of oligomers of MCU located in the IMM formed by two transmembrane domains, where the C- and N-termini point to the mitochondrial matrix [171]. Among the regulatory subunits, mitochondrial calcium uptake protein 1 (MICU1) exerts a gatekeeper function, stabilizing the closed state of the MCUC, thus allowing Ca^2+^ accumulation in the mitochondrial matrix but inhibiting mitochondrial Ca^2+^ entry [172]. MICU2, which has a 25% identity with MICU1, interacts with both MICU1 and MCU, and its function is still debated [173]. EMRE, an efflux multidrug resistance protein, is a single-pass membrane member of the complex that functions as a bridge between MICU1 and MCU, and its loss causes the same reduction in mitochondrial Ca^2+^ uptake as MCU depletion [174]. MCUb is an MCU isogene that acts as an endogenous dominant-negative isoform [175]. Finally, mitochondrial calcium uniport regulator 1 (MCUR1) enhances MCU activity by directly interacting with MCU but not with MICU1 [176].

Mitochondrial Ca^2+^ overload due to MCU activation causes mPTP opening and the release of factors that initiate necrosis and/or apoptosis [151,177]. When cancer cells are treated with proapoptotic stimuli, the expression of MCU relates to the sensitivity to these treatments [178]. Moreover, it seems that a chronic increase in mitochondrial Ca^2+^ load through MCU leads to mitochondrial stress and fragmentation of the mitochondrial network [167].

Regarding the implication of MCU in chemoresistance, there is evidence that in human colon cancer, microRNA-25 (miR-25) is able to target and downregulate MCU expression, decreasing mitochondrial Ca^2+^ uptake and favoring cancer cell proliferation and resistance towards proapoptotic stimuli since the reintroduction of MCU sensitizes cancer cells to the treatment [179,180]. On the other hand, a recent study demonstrated that the overexpression of receptor-interacting protein kinase 1 (RIPK1) and its consequent interaction with MCU is able to increase mitochondrial Ca^2+^ uptake, resulting in an increase in the proliferation of cancer cells [181].

Additionally, other evidence shows that in HCC, the expression of MCUR1 is increased and is correlated with an increase in MCU activity leading to greater mitochondrial Ca^2+^ uptake, which enables desensitization to proapoptotic stimuli [182].

In breast cancer patients, it has been demonstrated that survival rate is negatively correlated with an increase in MCU expression and a decrease in MICU1 expression, suggesting that MICU1 might act as an oncosuppressor [183]. Other evidence shows that the downregulation of MICU1 and MICU2, which occurs in pancreatic cancer cells, is facilitated by histidine triad nucleotide-binding protein (HINT2), whose decreased expression correlates with a poor prognosis and chemoresistance [184]. Furthermore, MICU1 knockout cells lose MCU gatekeeper function and become more susceptible to apoptotic and stress stimuli since oxidative stress and ROS production increase during mitochondrial Ca^2+^ uptake [185].

Enhanced MICU1 expression has been found in many types of cancers, and it is related to poor clinical outcomes and increased glycolysis and chemoresistance. Recent evidence shows that miR-195 targets the MICU1 3′-untranslated region (UTR), lowering its expression; in fact, stable miR-195 expression in ovarian cancer human xenograft models significantly reduces tumor growth and enhances cell survival. Thus, miR-195, which regulates MICU1, can be exploited to normalize abnormal MICU1 expression and reverse chemoresistance [186].

Altogether, both MCU and its negative regulator MICU1 may be considered important potential targets for combating cancer chemoresistance [187,188,189].

#### 3.1.2. Voltage-Dependent Anion Channels (VDACs)

Mitochondrial porins are also known as VDACs, and they reside in the OMM and take part in MOMP upon apoptotic stimulation. The porin family includes three isoforms named VDAC1, VDAC2, and VDAC3 [190]; the first two isoforms are the most involved in MOMP [191,192,193,194]. VDAC1 mediates the flow of small molecules, such as ROS, ATP, Ca^2+^ ions, and water molecules, across the OMM, making it crucial for metabolic signaling and Ca^2+^ signaling under physiological conditions [167]. This isoform is expressed in many types of cancers [192] and plays an essential role in tunneling ATP across the OMM directly to the first enzyme of glycolysis, hexokinase, whose expression is increased in cancer cells helping to maintain the Warburg effect [195,196,197].

The importance of VDAC1 has also been reported in chemoresistant cancer patients since a truncated but channel-forming isoform of VDAC1 (VDAC1-ΔC) has been detected in late-stage tumor tissue and tissues from chemoresistant lung adenocarcinoma patients; these findings demonstrate that VDAC1-ΔC is induced by HIF-1 in hypoxic conditions, conferring protection from apoptosis [198,199,200].

Another study highlighted that dexamethasone-resistant childhood acute lymphoblastic leukemia (ALL) patients showed lower expression levels of VDAC1 than healthy controls; hence, VDAC1 might be considered a predictor of the chemotherapy response for childhood ALL [201].

Regarding VDAC2, high transcript levels were found to be associated with a greater risk of tumor recurrence and resistance to hormonal therapy in high-risk breast cancer patients [202].

Furthermore, VDAC1 anchors to antiapoptotic BCL-2 and BCL-XL proteins, where BCL-2 is able to decrease VDAC1 channel conductance by possibly binding directly to it. Indeed, synthetic peptides corresponding to the VDAC1/BCL-2-interacting region decrease protection against staurosporine-induced apoptotic cell death in BCL-2-overexpressing cells, suggesting that a VDAC1-based peptide may prevent BCL-2 binding to the OMM, thus potentiating the efficacy of chemotherapy [203]. Intriguingly, the efficacy of VDAC1-based peptides has been proven over the years in different cancers by preclinical models [204]. It has been reported that these peptides can prevent the interaction with hexokinase II, BCL-XL, and BCL-2 in in vivo models [205,206].

Then, the great challenge to overcoming VDAC-mediated chemoresistance would be the design of specific VDAC1 inhibitors since it has been reported that VDACs are “druggable” channels [187,207] and isoform-specific inhibitors are so far unavailable.

#### 3.1.3. Na^+^/Ca^2+^/Li^+^ Exchanger (NCLX)

The majority of mitochondrial Ca^2+^ extrusion is mediated by NCLX, and the proper balance between Ca^2+^ uptake by MCU and Ca^2+^ extrusion by NCLX has a critical role in the maintenance of mitochondrial Ca^2+^ homeostasis, cell metabolism, and cell fate in general [208]. Although mitochondrial Ca^2+^ homeostasis alteration can be considered a cancer hallmark, the details of its role in the regulation of cancer progression, metastasis and chemoresistance are still poorly understood.

In the previous section, we discussed the role of MCU in cancer progression and chemoresistance and how the altered expression of its components or of MCU itself increases mtCa^2+^ intake with diverse outcomes in terms of cancer cell progression. The NCLX-mediated process, however, occurs approximately 100-fold slower than that of MCU; thus, NCLX function is a rate-limiting factor in the regulation of mitochondrial Ca^2+^ homeostasis [208,209].

Although NCLX plays an essential role, its role in cancer biology has not yet been fully investigated.

A recent work by Pathak and collaborators demonstrated that NCLX loss decreases mitochondrial Ca^2+^ extrusion and, as a consequence, inhibits proliferation and primary tumor growth while enhancing metastasis and chemoresistance in colorectal cancer. This dichotomous and apparently contradictory role of NCLX is supported by evidence that a decrease in its expression is accompanied by the upregulation of genes involved in epithelial-mesenchymal transition (EMT), cancer stemness, and downregulation of cell cycle progression mediator genes. These changes result in mitochondrial Ca^2+^ overload, membrane depolarization, and increased mitochondrial ROS production, which enhances the mesenchymal phenotype, driving colorectal cancer towards metastatic dissemination and treatment resistance [210].

### 3.2. Soluble Mitochondria-Related Proteins

Other than the role exerted by the proteins located at the IMM or the OMM, it is known that there are soluble cytoplasmic proteins able to modulate mitochondrial plasticity and Ca^2+^ homeostasis in many pathophysiological settings. In recent years, researchers have focused on proteins that can locate, under certain conditions, at MAMs to regulate the function of channels or receptors resident in the ER or mitochondrial membranes. Nevertheless, these proteins that are normally soluble in the cytoplasm are able to migrate to these membranes, regulating intracellular Ca^2+^ homeostasis in many pathological conditions, such as cancer, inflammation, diabetes, and neurodegeneration [24,25,211].

In the next section, we will focus on the role of some of the soluble proteins, other than the ones that migrate to MAMs, whose involvement in the regulation of mitochondrial Ca^2+^ homeostasis has been linked to the chemoresistant phenotype in cancer cells or patients. The current findings suggest that not only mitochondria isolated from cells but also mitochondria-associated soluble proteins may have dynamic action that might be crucial in the understanding of the drug resistance condition.

#### 3.2.1. S100A8

S100A8 is a part of the 22-member calcium-binding EF hand-containing superfamily, and it is a multifunctional protein able to heterodimerize with S100A9 to form calprotectin involved in the sequestration of divalent cations, acting mainly in the regulation of the innate immune system [212,213].

According to the literature, S100A8 is involved in inflammation, cell proliferation, and oncogenesis [214]; indeed, S100A8 has been reported to be associated with apoptosis, autophagy, myeloid differentiation, and chemotherapy resistance [215,216,217]. Moreover, S100A8 is widely expressed in many tissues and cells, but it is particularly abundant in myeloid cells [218].

Some studies have reported that S100A8 has a role in mitophagy promotion in cancer cells related to the crosstalk between mitochondria and lysosomes mediated by ROS or via the activation of the autophagy initiation complex BECN1-PI3KC3 (see mitophagy section in this review for further details) [215,217].

Furthermore, autophagy and mitophagy have been ascertained to play an essential role in chemotherapy resistance [219]. Indeed, the inhibition of autophagy may promote sensitivity to chemotherapeutic treatment and apoptosis in many types of malignant cancers [220]. Moreover, multiple chemotherapeutic drugs are able to increase the autophagic/mitophagic flux by promoting the drug resistance phenotype and cell survival [221,222].

In a recent work by Zhang and coworkers, the relationship between S100A8, mitophagy, and chemotherapy resistance in B-cell lymphoma cells were characterized. They reported that S100A8 increased drug resistance through the stimulation of BECN1-PI3KC3 and BECN1-BCL-2 complex formation, prompting early mitophagic signaling. In addition, S100A8 enhanced BNIP3 expression, boosting mitophagic signaling activation [223]. BNIP3 is a 19 kDa BH3-only protein located at the mitochondria-ER interface; specifically, it is anchored to the OMM through the C-terminal domain, while its N-terminal domain points to the cytoplasm. BNIP3 is an autophagy and mitophagy inducer (see the paragraph) and triggers cell death by affecting mitochondrial function [217,224,225,226]. It has been reported that its aberrant expression is related to mitochondrial Ca^2+^ homeostasis and promotes cell death [100,227].

Thus, S100A8, a small Ca^2+^-binding protein, might represent an intriguing link between chemoresistance, mitochondrial Ca^2+^ homeostasis, and mitophagy.

#### 3.2.2. Sorcin

Soluble resistance-related calcium-binding protein (Sorcin) is a 22 kDa soluble protein belonging to the Penta EF-hand (PEF) protein family. Basically, Sorcin works as a Ca^2+^ sensor in the cytoplasm, as its role was firstly characterized in cardiomyocytes. Indeed, it takes part in the relaxation process after excitation-contraction coupling (EC coupling), by restoring [Ca^2+^]_i_ to resting conditions [228,229] and by activating SERCA pumps at the ER, voltage-dependent L-type Ca^2+^ channels, and Na^+^/Ca^2+^ exchangers (NCXs) at the plasma membrane, and possibly MCU at mitochondria while inhibiting the ryanodinic receptor (RyR) at the ER [230,231,232,233,234].

In addition to its role in the regulation of intracellular Ca^2+^ homeostasis, as the name itself suggests, it has been found to be widely overexpressed in many cancer types, especially in chemotherapy-resistant specimens [235,236,237,238,239]. In many cases, the chemoresistant phenotype was a result of Sorcin co-amplification with *mdr1*, a gene coding for an ATP-binding cassette pump considered a biomarker of MDR since its overexpression facilitates the extrusion of drugs from cells [240,241,242,243,244,245]. Moreover, it has been demonstrated that Sorcin overexpression is related to a poor clinical outcome in leukemia patients [246,247], and the combination of Sorcin silencing or depletion and chemotherapy treatment improves the effectiveness of treatment and the sensitivity to death stimuli [248,249,250,251,252,253,254].

In addition, it has been reported that a shorter isoform of Sorcin (18 kDa) interacts with TRAP1, a mitochondrial chaperone (Hsp75) with antioxidant and antiapoptotic functions, in mitochondria. TRAP1 exerts a role in MDR in cancer cells and is upregulated together with Sorcin in colorectal carcinoma cells; moreover, their interaction is required for Sorcin localization at mitochondria and TRAP1 stability [255]. TRAP1 and Sorcin are co-upregulated in cancer, and this feature is considered a marker of drug resistance, but their reciprocal regulation also relates to TRAP1 translational control of Sorcin; indeed, TRAP1 silencing relates to Sorcin protein instability and degradation [255]. Moreover, this quality control exerted by TRAP1 on Sorcin protects against apoptosis either induced by ER stress pathway activation or paclitaxel administration in breast carcinoma [256].

A recent publication also showed that Sorcin silencing increases mitochondria-ER proximity, while overexpression decreases the proximity with an effect mainly mediated by cytosolic [Ca^2+^] alteration [257]. This evidence indicates that the Sorcin-mitochondria relationship is a crucial feature in the regulation of cancer cell survival and chemotherapy resistance, suggesting a potential role of proto-oncogenes as well as an intriguing therapeutic target since Sorcin links Ca^2+^ homeostasis to the mitochondrial response to stress stimuli, which is related to drug resistance.

#### 3.2.3. PKCζ

Protein kinase C (PKC) is part of a protein family that comprises serine/threonine kinases that are involved in a large number of signaling pathways from cell proliferation to apoptosis and from muscle contraction to secretion [258,259,260]. The members of this protein family have been subdivided into three classes: (i) conventional PKCs (isoforms α, β1, β2, and γ) activated by diacylglycerol and Ca^2+^, (ii) novel PKCs (δ, ε, η, and θ) activated by diacylglycerol and Ca^2+^-independent, and (iii) atypical PKCs (λ and ζ) that are both diacylglycerol- and Ca^2+^-independent [261]. Interestingly, Ca^2+^ activation of some PKCs and PKC kinases affects the spatiotemporal pattern of the Ca^2+^ cellular response since it has been noted that PKCs can modulate agonist-mediated Ca^2+^ release from the ER and differentially decode low- and high-frequency Ca^2+^ spikes [262,263].

Among the isoforms, PKCζ is implicated in either apoptotic or mitogenic signals as it acts on different pathways. These characteristics provide important implications for PKCζ in tumorigenesis [264,265,266,267,268]. Moreover, it has been reported that PKCζ acts as an antiapoptotic factor reducing cancer cell sensitivity to chemotherapeutic treatment [269,270] and is possibly implicated in linking cancer-related inflammation and chemoresistance via NF-kB activation and nuclear translocation [271]. Indeed, there is evidence that upon oxidative stress, PKCζ is able to translocate to the nucleus and to increase resistance to apoptotic inducers; in turn, a recombinant nuclear PKCζ inhibitor restores the sensitivity towards apoptotic stimuli in chemoresistant cells [272].

It has also been demonstrated that PKCζ, similar to some other PKCs, is linked to the regulation of mitochondrial Ca^2+^ concentration upon agonist stimulation. Specifically, this isoform increases mitochondrial uptake of Ca^2+^ upon histamine stimulation with no significant variation in the increase in ROS production or ΔΨ, which can be considered the Ca^2+^ driving force in the mitochondria; no significant changes in either cytosolic or ER Ca^2+^ concentration occur during this process [273].

Although it is mainly soluble and expressed in the nucleus, PKCζ affects mitochondrial [Ca^2+^] with possible implications in cancer progression regulation and multidrug resistance in a way that has not yet been fully disclosed. Thus, the understanding of the signaling pathways in which PKCζ lies at the crossroads between mitochondrial Ca^2+^ regulation and chemoresistance would be extremely helpful and interesting in the development of novel therapeutic strategies.

## 4. Oxidative Stress and Bioenergetic Remodeling in Chemoresistance

In 1954, Gerschman and his group first theorized about the toxic effects of oxygen due to partially reduced forms known as free radicals [274], generated as a byproduct of metabolic processes. ROS include the superoxide anion (O_2_^−^), singlet oxygen (^1^O_2_), hydrogen peroxide (H_2_O_2_), and hydroxyl radical (HO·). Free radicals also include reactive nitrogen species (RNS). These unstable and partially reduced oxygen derivatives act as second messengers in cell signaling. Within the cell, they play a double role since they have both beneficial and deleterious effects. On the one hand, at low doses, they are involved in intracellular signaling and adaptive and innate immune responses. On the other hand, high levels lead to biological damage known as oxidative stress as a result of an imbalance between ROS production and antioxidant defense systems [275,276]. Mitochondria represent the major source of ROS, producing almost 90% of total ROS as a consequence of OXPHOS [277]. It is well recognized that ROS accumulation can cause direct damage to organelles and biomolecules (DNA, proteins, and lipids), which, through an inflammatory response, may lead to cancer development [278]. However, to counterbalance ROS overproduction, cells adopt not only enzymatic antioxidants, such as superoxide dismutase (SOD), catalase (CAT), glutathione peroxidases (GPxs), and thioredoxin (Trx) but also nonenzymatic antioxidants, which jointly minimize oxidative stress [279].

In cancer cells, an increased metabolic rate, the dysfunction of mitochondria, and activation of oncogenes (e.g., c-Myc, Kras, and BRCA1) are thought to be some of the factors responsible for ROS production [280,281,282,283].

ROS support tumor cells in several cancer-related mechanisms, such as survival, angiogenesis, and metastasis [284,285,286]. Interestingly, conflicting roles of ROS as crucial secondary messengers in cancer and during cancer chemotherapy have emerged [286,287]. Indeed, a growing body of evidence supports the role of ROS not only as a tumor promoter but also as a tumor suppressor [288] in view of the fact that most chemotherapy, radiotherapy, and photodynamic therapy approaches increase intracellular levels of ROS to trigger cancer cell death [289].

In this context, anthracyclines, such as doxorubicin, daunorubicin, and epirubicin, produce the highest ROS levels [290]. Platinum complexes, alkylating agents, camptothecins, and topoisomerase inhibitors also induce the production of high amounts of cellular ROS [291,292,293]. In contrast, vinca alkaloids, taxanes, and antimetabolites (antifolates and nucleosides) lead to lower levels of ROS [289]. Mitochondrial ROS generation and inhibition of the antioxidant system represent the main reasons for elevated ROS levels. For instance, arsenic trioxide, used in leukemia treatment, provokes mitochondrial membrane potential reduction and inhibition of complexes I and II of the electron transport chain (ETC), triggering ROS overproduction [294,295].

Imexon, a prooxidant small molecule, binds to glutathione (GSH) and cysteine, causing a decrease in the level of cellular GSH and causing the accumulation of ROS in patients with metastatic cancer [296]. The anticancer activity and safety of imexon in leukemia have been confirmed in preclinical and phase I/II clinical trial studies [297]. Mangafodipir inhibits SOD, leading to an increase in H_2_O_2_ levels that triggers apoptosis in cancer cells [298].

Cisplatin, one of the most effective and widely used chemotherapeutic drugs, is known to provoke DNA adducts that, if not repaired, cause DNA damage, leading to ROS generation [299]. Interestingly, Marullo et al. demonstrated that exposure to cisplatin stimulates a mitochondrion-dependent ROS response that boosts its cytotoxic effect towards cancer cells [300].

Even though most chemotherapeutic drugs increase ROS to cytotoxic levels, in cancer cells, such ROS exposure may also minimize chemotherapy effects in the long term, thereby causing chemoresistance [287,301,302].

Notably, resistance to chemotherapeutic drugs is among the leading causes of cancer-related death [303]. It has been reported that 2-deoxy-D-glucose (2-DG) is able to promote chemoresistance through ROS-stimulated upregulation of P-glycoprotein expression (P-gp) [304,305]. In addition, 2-DG may also induce chemoresistance in human ovarian and breast cancer cells by upregulating the expression of dihydrodiol dehydrogenases (DDHs) [306]. Rimessi et al. demonstrated that oxidative stress triggers PKCζ accumulation within the nucleus and reduced the sensitivity of cancer cells to chemotherapeutic agents, confirming that this PKC isoform may serve as a useful target for tumor cell chemosensitization [272] (Table 1).

Tumor heterogeneity plays a key role in chemotherapeutic drug resistance [301]. Given that, heterogeneity has been noted to be tightly correlated with high levels of ROS [307].

However, ROS involvement in tumor heterogeneity still requires further investigation. In this scenario, increasing evidence suggests that CSCs co-occur, at least in part, in the emergence of cancer heterogeneity [301].

Especially in recent decades, CSCs have gained growing attention since they are a subset of cancer cells with stemness characteristics that have been detected in several tumors, such as leukemia [308], breast cancer [309], and pancreatic cancer [310]. For this reason, CSCs appear to be responsible for cancer recurrence after chemotherapy or radiotherapy [311].

In murine and human breast, CSCs have been detected to have a lower level of ROS than their corresponding nontumorigenic cells, which was correlated with higher expression of free radical scavenging system components. Hence, this indicates that enhanced ROS defenses may be responsible for tumor radioresistance [312].

Similarly, Phillips et al. demonstrated that breast CSCs were radiotherapy-resistant and displayed low levels of ROS [313].

Hence, it would be advantageous to develop new delivery strategies, such as nanoparticle delivery systems, to be applied in the clinic to boost and maintain ROS levels for a certain period of time to reverse drug resistance [287].

In the context of chemoresistance, metabolic reprogramming has gained substantial consideration and is now considered a cancer hallmark [314]. These metabolic alterations, some more glycolysis-oriented and some more oxidant-oriented are adopted by cancer cells to overcome stress conditions such as hypoxia or limited nutrients. In addition, these different bioenergetic features may coexist within the same tumor mass (tumor heterogeneity), leading to different responses to chemotherapy [315]. In addition to glycolysis and OXPHOS, it is well documented that most cancer cells are also avidly dependent on glutamine supply, which is referred to as “glutamine addiction” [316,317].

For years, tumor cells have been considered to be more reliant on glycolysis (the “Warburg effect”) than normal cells and are characterized by dysfunctional mitochondria [318].

However, in recent years, the importance of OXPHOS in many cancer settings has been increasingly reported, highlighting the different metabolic requirements of cancer cells [319].

High-grade serous ovarian cancer (HGSOC) is one of the most aggressive ovarian cancers and has been reported by Gentric and collaborators to display the highest metabolic heterogeneity. They identified two distinct cellular subgroups, low OXPHOS (relying on glycolysis) and high OXPHOS (relying on OXPHOS, glutamine, and fatty acid oxidation). Interestingly, high-OXPHOS HGSOC exhibited chronic oxidative stress that stimulated the activation of PGC1α. Active PGC1α enhanced the synthesis of ETC complexes, promoting mitochondrial respiration and thus leading to an increased response to conventional chemotherapies [320]. In contrast, in another study, it was reported that cisplatin-resistant HGSOC cells relied on OXPHOS more than their sensitive counterparts and that inhibition of OXPHOS restored cisplatin sensitivity [321].

Furthermore, it has been demonstrated that reduced expression of the mitochondrial chaperone tumor necrosis factor-associated protein (TRAP1), which is known to have a pivotal role in metabolic rewiring, mediates a shift towards OXPHOS, which causes ovarian cancer resistance to cisplatin treatment [322].

Conversely, in a recent study, it was demonstrated that in ovarian cancer cells, MICU1 increases aerobic glycolysis and thus chemoresistance [323].

Additionally, melanomas exhibit unexpected metabolic features. Indeed, it has been reported that this highly aggressive type of cancer develops alternative metabolic strategies to survive and proliferate [324]. Lim and coworkers demonstrated that a subset of human melanomas depend on OXPHOS and that PGC1α overexpression in these cells facilitates resistance to oxidative stress. Intriguingly, the inhibition of PGC1α triggers ROS overproduction, HIF1α stabilization, and a metabolic switch towards glycolysis. Subsequently, suppression of both PGC1α and HIF1α causes energetic defects that lead to the emergence of a compensatory mechanism based on the use of glutamine to survive.

Hence, this evidence demonstrates that three alternative metabolic strategies ensure tumor resistance and survival and that combinatorial therapy is required [324].

Similarly, HCC, pancreatic, and colon cancer cells rely on OXPHOS to survive [325,326,327]. Hence, the usage of OXPHOS inhibitors could be a promising strategy to overcome tumor resistance [326]. As mentioned above, oxidative stress and metabolic remodeling of cancer cells are strongly associated with cancer chemoresistance, in which mitochondria play a fundamental role. Combinatory therapeutic approaches aimed at targeting different metabolic pathways, such as glycolysis, OXPHOS, and even glutaminolysis, could represent a promising strategy for preventing chemoresistance. In addition, exploiting ROS-modulating treatment to eliminate cancer cells and CSCs could strengthen the efficacy of traditional anticancer therapies.

## 5. Conclusions

Chemoresistance, as a survival strategy engaged by cancer cells upon apoptotic stimulation, is inevitably connected to mitochondrion-related pathways. Mitochondria are essential players in cancer cell survival, as they play roles in processes from metabolism to Ca^2+^ signaling and from mitochondrial dynamics regulation to oxidative stress. Since successful therapies are still needed, it is important to obtain a deeper knowledge of the connections between the players of mitochondria-related pathways.

In fact, current evidence indicates that mitochondria dynamics, which can be both positive and negative regulators of chemoresistance depending on activation timing, cancer cell type, and most importantly, tumor microenvironment, can be considered a potent regulator of cancer drug resistance and thus are potentially crucial targets.

In addition, metabolism-targeted approaches centered on OXPHOS, glutamine metabolism, or ROS might represent successful strategies for inhibiting both cancer proliferation and drug resistance.

Mitochondrial Ca^2+^ signaling, which is directly modulated by proteins embedded either in the OMM or IMM and/or cytosolic proteins associated with mitochondria, might represent other compelling targets for successful cancer treatment. However, broader characterization of these proteins and their relationship to tumorigenesis and cancer drug resistance through mitochondrial Ca^2+^ homeostasis regulation is still required.

Taken together, the advancements in the characterization of tumorigenesis and chemoresistance in the context of mitochondrial functionality are not only interesting but also crucial to the understanding of cancer mechanisms and for designing optimal therapeutics against uncontrolled cell survival and resistance to apoptotic stimuli.

## Figures and Tables

**Table 1:** Effects and targets of chemotherapeutic drugs on mitochondrial and cellular oxidative stress.

Chemotherapeutic Drug	Target	Effect	References
Anthracyclines: Doxorubicin, Epirubicin, and Daunorubicin	Topoisomerase II	↑ ROS	[290]
Doxorubicin	Electron transport system (ETS)		[289]
Alkylating agents, camptothecins, and topoisomerase inhibitors	DNA topoisomerase I	↑ ROS	[291,292,293]
Vinca alkaloids, Taxanes, and Antimetabolites (antifolates and nucleoside)	Cytoskeleton, β-tubulin	↓ ROS	[289]
Arsenic trioxide	Complexes I and II of the ETC	↓ ΔΨm ↑ ROS	[294,295]
Imexon	GSH and cysteine	↓ GSH ↑ ROS	[296]
Mangafodipir	SOD	Increase in H_2_O_2_ levels that trigger apoptosis	[298]
Cisplatin	DNA	DNA adducts, and ROS generation	[299]
2-deoxy-D-glucose (2-DG)	↑ P-gp ↑ DDHs	Chemoresistance ↑ ROS Chemoresistance	[304,305,306]

↑: represents the increased production, in the case of ROS, or increased expression levels of P-gp or DDHs. ↓: represents the decrease in ROS production or mitochondrial membrane potential (ΔΨm), or decreased expression levels of GSH.
